# Cervical Cancer Screening Service Utilization and Associated Factors among Women in the Shabadino District, Southern Ethiopia

**DOI:** 10.1155/2020/6398394

**Published:** 2020-07-03

**Authors:** Jeylan Kasim, Abdurehman Kalu, Bekele Kamara, Haileselasie Berhane Alema

**Affiliations:** ^1^College of Health Sciences and Referral Hospital, Madda Walabu University, Goba, Ethiopia; ^2^Southern Nations Nationalities and People Region Health Bureau, Shabadino District Hospital, Sidama, Ethiopia; ^3^Department of Public Health, College of Health Sciences, Aksum University, Axum, Ethiopia

## Abstract

**Background:**

Cervical cancer is the major cause of morbidity and mortality among women worldwide with an estimated 528,000 new cases and 266,000 deaths annually. In Ethiopia, there are 7095 new cases and 4732 deaths of cervical cancer every year. But cervical cancer screening utilization remains limited. Therefore, the aim of the study was to assess cervical cancer screening utilization and associated factors among women in the Shabadino district, Southern Ethiopia.

**Methods:**

A community-based cross-sectional study was conducted in the Shabadino district, Southern Ethiopia, using a structured questionnaire. A systematic random sampling method was used to recruit 536 study participants. The collected data were entered and analyzed using SPSS version 22.0. Bivariate and multivariate logistic regressions were used to assess factors associated with cervical cancer screening utilization at a 95% level of significance and a *p* value of less than 0.05.

**Results:**

The study revealed that among 506 women, only 52 (10.3%) have been screened for cervical cancer. Women who are educated (completed primary school and above) (AOR = 1.9; 95% CI = 1.18-3.05), who have a history of the presence of sexually transmitted diseases (AOR = 2.6; 95% CI = 1.26-5.23), who have multiple sexual partners (AOR = 4.0; 95% CI = 1.86-8.66), and who knew methods of cervical cancer prevention (AOR = 4.3; 95% CI = 1.18-13.05) were significantly associated with high cervical cancer screening utilization.

**Conclusion:**

The magnitude of cervical cancer screening utilization among women was very low. Educational status, history of multiple sexual partners, history of sexually transmitted diseases, and knowing methods of prevention were significant factors of high cervical cancer screening utilization. *Recommendation*. It is very crucial to implement an appropriate awareness creation method. Additionally, the STI clinic should be linked to the cervical cancer screening service to increase the knowledge of cervical cancer prevention and the utilization of cervical cancer screening.

## 1. Background

Cervical cancer is one of the gravest threats to women's lives. Worldwide, currently, it is estimated that over a million women have cervical cancer. Most of these women have not been diagnosed, nor they have access to treatment that could cure them or prolong their lives [1].

Cervical cancer is the fourth most common cancer in women, and seventh overall, with an estimated 528,000 new cases worldwide. A majority (around 85%) of the global burden occurs in the less developed regions. There are about 266,000 deaths from cervical cancer worldwide that accounts for 7.5% of all female cancer deaths. Almost nine out of ten (87%) cervical cancer deaths occur in less developed regions [2].

In sub-Saharan Africa, 34.8 new cases of cervical cancer are diagnosed and 22.5 die per 100,000 women annually [3]. In Ethiopia, cervical cancer ranks the second most common type of cancer among women. Every year, 7095 women are diagnosed with cervical cancer, and 4732 die from the disease [4, 5]. Though there were only a few studies in Ethiopia, the cervical cancer screening service utilization result ranged from 4.8% to 19.8% [6–8].

According to the American Cancer Society Report, risk factors for cervical cancer include sexual intercourse at an early age, multiple sexual partners, tobacco smoking, long-term oral contraceptive use, low socioeconomic status, immunosuppressive therapy, and micronutrient deficiency [9].

The main strategies of cervical cancer prevention are immunization of human papilloma virus (HPV) vaccine and screening for cervical cancer to detect and remove cancerous lesions. Regular screening and early treatment highly decrease the incidence of cervical cancer. To reduce the incidence and mortality associated with the disease, early screening and treatment as part of targeted interventions is mandatory. So, concrete evidence is crucial for the development of strategies, policy, and planning. But there are very limited studies conducted in the country to assess the utilization of cervical cancer screening. Therefore, this study is aimed at assessing cervical cancer screening service utilization and associated factors among women of 30-49 aged in the Shabadino district, Ethiopia.

## 2. Methods

### 2.1. Study Settings

This study was conducted in the Shabadino district, Sidama zone, Southern Ethiopia. The district comprises 35 rural kebeles. It is 27 kilometers way from Hawassa, the capital city of the southern region. As shown from the estimated projection of 2007 Central Statistical Agency [10], the district has a total population of 267,487. From this population, 131,068 (49.3%) are males and 136,419 (50.7%) are females. The district has a total of 54,587 households. There are 45 health facilities in the district which include one district hospital, nine health centers, and 35 health posts. But from health facilities, only one health center offers cervical cancer screening services [11]. The community-based cross-sectional study design was conducted from February to March 2018.

### 2.2. Study Population

The study population included women whose age ranged from 30 to 49 years in the Shabadino district in the past one year, whereas women with a history of cervical and/or uterus removal and who are positive for cervical cancer were excluded.

### 2.3. Sample Size Determination

The sample size was determined using a single population proportion formula based on assumptions of 95% confidence interval, utilization of cervical cancer screening from a previous similar population study, which was 19.8 [6] and with marginal error of 5%.

(1) $$\begin{array}{c} n=\frac{{\left(Z\alpha /2\right)}^{2}p\left(1-p\right)}{d^{2}.}. \end{array}$$

The sample size was 244, and due to the multistage sampling technique, the design effect applied by multiplying with 2 and 10% of nonresponse rate was added to get the final sample size of 536.

### 2.4. Sampling Procedure

Among the total 35 kebeles found in the Shabadino district, 11 kebeles were selected by a simple random sampling technique. Then, 536 households were selected using a systematic random sampling technique and the total sample size was allocated proportionate selected kebele to the size of their households.

Based on the national plan of 2007, the projected estimate of the district comprised 10,568 households in eleven selected kebeles. Therefore, the sampling interval of households in each kebele was determined by dividing the number of households to the allocated sample size of the respective kebeles. The initial household was selected randomly by a lottery method. The subsequent households included in the study were identified by systematic random sampling through house-to-house visit after nominating and adding the sample interval to each household with the pervious number till the total number of sample size was achieved. If there was a household with more than one eligible woman, one woman was randomly selected using the lottery method. Then, the objective of the study was explained by the data collector and consent was asked to participate in the study.

### 2.5. Data Quality Assurance

The questionnaire was prepared in English and translated to “SidamuAfoo” and retranslated to check its consistency. All were trained for three days on the objective of the study, the contents of the questionnaire, the issues related to the confidentiality of the response, and the right of the respondents. Close supervision was conducted during the process of data collection, and questionnaires were checked for consistency and completeness.

### 2.6. Data Processing and Analysis

After data collection was completed and questionnaires were edited and coded, the data were entered into a computer and processed by using the Statistical Package for the Social Sciences (SPSS) version 22.0 for further analysis. Descriptive statistics like frequency, percentage, and mean with standard deviation were used to describe the study population in relation to relevant variables. Binary logistic regression analysis with a 95% confidence interval was used to assess the eligible variable for the multivariable analysis model. And variables that had a significant association with the outcome variable at *p* value less than 0.2 were entered into the multivariate analysis model. Adjusted odds ratio (AOR) with a 95% confidence interval (CI) and *p* value of less than 0.05 were used to identify factors associated with the utilization of cervical cancer screening.

### 2.7. Operational Definitions

#### 2.7.1. Cervical Cancer Screening Utilization

Cervical cancer screening utilization was assessed by asking the respondent's action towards screening for cervical cancer within one year. Those who screened within the past one year were categorized as having *utilized*, and those who never screened were labeled as having *not utilized* [12].

#### 2.7.2. Good Knowledge

The scores of the knowledge base item questions were computed, and a score greater than the mean score was considered *good knowledge* whereas below the mean score is *poor knowledge* [13].

## 3. Results

### 3.1. Sociodemographic Characteristics of Respondents

From a total of 536 selected eligible women, 506 have participated in the study with the response rate of 94%. The mean age of participants was 37 (± 5.3 SD) years (Table 1).

### 3.2. Sexual and Reproductive Characteristics of Respondents

Almost all (503, 99.4%) of the participants had sexual intercourse, and among them, 430 (85%) had their first sexual intercourse at the age of 18 and above (Table 2).

### 3.3. Knowledge and Prevalence of Cervical Cancer Screening Utilization

From the study participants, 321 (63.4%) had heard about cervical cancer. Of those who had heard about cervical cancer, 293 (58%) knew about its risk factors, 215 (42.5%) knew about its symptoms, and 150 (29.5%) knew about its methods of prevention.

Concerning their source of information about cervical cancer, mass media was the most common source of information preceding by health workers and health extension workers. Two hundred two (39.9%) of the respondents have good knowledge, and 304 (60.1%) have poor knowledge about cervical cancer prevention.

The prevalence of cervical cancer screening utilization was found to be 52 (10.3%).

### 3.4. Factors Associated with Cervical Cancer Screening Utilization

On bivariate analysis, factors found to be significantly associated with utilization of cervical cancer screening service were educational status, household monthly income, hearing about cervical cancer, history of the sexually transmitted disease, HIV test, multiple sexual partners, knowing the causes of cervical cancer, and knowing the prevention methods of cervical cancer.

After controlling for confounders using the multivariate analysis model, educational status, history of sexually transmitted disease, and multiple sexual partners were significantly associated with the cervical cancer screening service utilization.

This study revealed that the educational level has a significant association with the cervical cancer screening utilization; women who attended primary education and above were about 2 times more likely to utilize cervical cancer screening service than those who had never attended any formal education (AOR = 1.89; 95% CI = 1.18-3.05, *p* = 0.009).

Women who have a history of sexually transmitted disease were about 2.6 times more likely to utilize cervical cancer screening when compared to those who have no history of sexually transmitted disease (AOR = 2.57; 95% CI = 1.26-5.23).

Women who have a history of multiple sexual partners were 4 times more likely to utilize cervical cancer screening when compared to those who have a single partner (AOR = 4.01; 95% CI = 1.86-8.66).

Regarding the knowledge status, women who knew that cervical cancer is a preventable disease were 4.3 times more likely to utilize cervical cancer screening service than those who do not know that cervical cancer is a preventable disease (AOR = 4.34; 95% CI = 1.18-13.05) (Table 3).

## 4. Discussion

This community-based study was conducted to assess the level of cervical cancer screening service utilization and its associated factors among women in the Shabadino district, Sidama Zone, Southern Ethiopia.

This study revealed that only 52 (10.3%) of respondents had screened for cervical cancer. This result was low compared to studies done in different parts of Ethiopia which were 15.5%, 16.5%, 22%, and 25% [14, 15, 12, 16]. The reason behind this is because the study populations are living in remote areas where they lack access to education and lack women empowerment. But the findings are somehow slightly higher from studies in Nigeria and Arba Minch town, Ethiopia, which were 8.0% [17] and 5.8% [7], respectively. This could be explained by the study period difference. Those studies were done some years back where awareness and availability of the service of cervical cancer screening were low.

The factors associated with the utilization of cervical cancer screening were educational status of the woman, presence of sexually transmitted diseases, presence of multiple partners, and knowledge of risk and prevention methods of cervical cancer.

Educational status was significantly associated with the utilization of cervical cancer screening. Women who attended primary school and above were 2 times more likely to utilize cervical cancer screening service compared to those who did not attend any formal education. This finding was consistent with the study done in Addis Ababa, Ethiopia [18], and Ghana [19]. Education can increase women's access to information from different sources. This could be due to the fact that educated women have better awareness of the benefit of cervical cancer screening service utilization.

The presence of sexually transmitted diseases was significantly associated with cervical cancer screening service utilization. Women who have a history of sexually transmitted diseases were 2.6 times more likely to be screened for cervical cancer than those who have no history of sexually transmitted diseases. This result is consistent with the study findings in different parts of Ethiopia [6, 20, 15].

Having multiple sexual partners was identified as a risk factor for cervical cancer screening utilization. Our study revealed that women who have a history of multiple sexual partners were 4 times more likely to utilize cervical cancer screening compared to those who have a single partner. The result is supported by studies done in Ethiopia and Malawi [6, 14, 21]. This is because women with multiple sexual partners may have a high risk of contracting sexually transmitted diseases (STDs). Once women develop symptoms for any sexually transmitted diseases, there is an increased chance of seeking medical care.

Knowledge on prevention methods of cervical cancer was found to be significantly associated with cervical cancer screening utilization. This study also showed that women with good knowledge of prevention methods of cervical cancer have a higher chance of cervical cancer screening utilization compared to those with poor knowledge. This result was similar to studies conducted in Hossana, Addis Ababa (Ethiopia), and Nigeria [12, 22, 23]. This is because women with good knowledge of prevention methods of cervical cancer had increased health care-seeking behavior. This makes them have a regular checkup for cervical cancer and other diseases to monitor their health status.

## 5. Limitation

This study involved a sensitive matter that may be subjected to social desirability bias. Meanwhile, the procedure to screen for cervical cancer and precervical lesion was similar for the women. The study participants may face difficulty to differentiate the type they are screened for.

## 6. Conclusion

The study revealed that the magnitude of cervical cancer screening service utilization among women was very low. Educational status, history of multiple sexual partners, presence of sexually transmitted diseases (STDs), and knowledge about prevention methods were significant factors associated with cervical cancer screening service utilization.

## Figures and Tables

**Table 1:** Sociodemographic characteristics of respondents in the Shabadino district, Sidama Zone, Ethiopia, 2018 (*n* = 506).

Variables	Frequency	Percentage (%)
Age group		
30-34	178	35.2
35-39	193	38.1
40-44	85	16.8
45-49	50	9.9
Religion		
Orthodox	45	8.9
Protestant	403	79.6
Muslim	54	10.7
Catholic	4	0.8
Ethnicity		
Sidama	447	88.3
Amara	42	8.3
Silite	7	1.4
Wolayita	10	2
Marital status		
Single	5	1
Married	468	92.5
Divorced	15	3
Widowed	18	3.5
Educational status		
Non-formal education	185	36.6
Primary education	239	47.2
Secondary school	64	12.6
College and above	18	3.6
Occupation		
Housewife	421	83.2
Self-employed	70	13.8
Government employee	15	3
Household monthly income		
<900	146	28.9
901-1600	258	51
1601-2700	83	16.4
>2700	19	3.7

**Table 2:** Sexual and reproductive characteristics of respondents in the Shabadino district, Sidama Zone, Ethiopia, 2018 (*n* = 506).

Variables	Frequency	Percentage (%)
Used COC pills		
No	327	64.6
Yes	179	35.4
Had history of STI		
No	387	76.5
Yes	119	23.5
HIV test		
No	126	24.9
Yes	380	75.1
Serostatus		
Negative	392	77.5
Positive	114	22.5
Had given birth		
No	8	1.6
Yes	498	98.4
Age at first sexual intercourse		
<18	89	17.6
≥18	417	82.4
Multiple sexual partners		
No	446	88.1
Yes	60	11.9

**Table 3:** Bivariate and multivariate analyses of factors associated with cervical cancer screening utilization among women in the Shabadino district, Sidama Zone, Ethiopia, 2018.

Variables	Cervical cancer screening utilization		COR (95% CI)	AOR (95% CI)	*p* value
	Yes	No			
Age group					
30-34	17	160	1		
35-39	22	171	1.14 (0.62-2.36)		
40-44	7	77	0.92 (0.34-2.15)		
45-49	6	46	0.77 (0.46-3.29)		
Educational status					
Non-formal education	9	191	1	1	
Primary education	20	212	2.00 (0.89-4.50)		
Secondary school	19	42	9.60 (4.06-22.70)	1.89 (1.18-3.05)^∗^	0.009
College and above	4	9	9.43 (2.44-36.54)		
Occupation					
House-wife	38	383	1		
Self-employed	9	62	1.46 (0.67-3.17)		
Governmental employee	5	9	5.6 (12)		
Had history of STI					
No	27	360	1	1	
Yes	25	94	3.55 (1.97-6.39)	2.57 (1.26-5.23)^∗^	0.021
Age at first sexual intercourse					
<18	27	621			
≥18	25	392	0.15 (0.08-0.27)		
Have history of multiple sexual partners					
No	414	32	1	1	
Yes	40	20	6.5 (3.39-12.34)	4.01 (1.86-8.66)^∗^	0.001
Knew the prevention methods of cervical cancer					
No	341	15	1	1	
Yes	113	37	7.4 (3.94-14.07)	4.34 (1.18-3.05)^∗^	0.001

^∗^Statistically associated with cervical cancer screening utilization at *p* value < 0.05.

## Data Availability

All the data supporting the findings is contained within the manuscript.

## Abbreviations

AOR: Adjusted odds ratio
COR: Crude odds ratio
CCPC: Cervical Cancer Prevention and Control
CIN: Cervical intraepithelial neoplasia
ETB: Ethiopian birr
FDRE: Federal Democratic Republic of Ethiopia
HICs: High-income countries
HPV: Human papilloma virus
IARC: International Agency for Research on Cancer
LMICs: Low- and middle-income countries
NCCPP: National Cervical Cancer Prevention Program
SPSS: Statistical Package for the Social Sciences
STI: Sexually transmitted infection
VIA: Visual inspection with acetic acid
VILI: Visual inspection with Lugol's iodine
WHO: World Health Organization.

## Additional Points

*Recommendation*. It is very crucial to implement appropriate awareness creation methods. All women in the STI clinic should be linked to screen for cervical cancer to increase the knowledge of cervical cancer prevention and the utilization of cervical cancer screening. The use of opportunities to provide health education about cervical cancer to women during antenatal care follow-up, during family planning service, and at STI clinic is vital.
